# Predictors of Life Expectancy in Community‐Dwelling People with Dementia – The Role of Social Support

**DOI:** 10.1002/alz.084646

**Published:** 2025-01-09

**Authors:** Iris Blotenberg, Melanie Boekholt, Bernhard Michalowsky, Moritz Platen, Francisca S Rodriguez, Stefan Teipel, Wolfgang Hoffmann, Jochen René Thyrian

**Affiliations:** ^1^ Deutsches Zentrum für Neurodegenerative Erkrankungen e. V. (DZNE), site Rostock / Greifswald, Greifswald Germany; ^2^ Deutsches Zentrum für Neurodegenerative Erkrankungen e. V. (DZNE), site Rostock / Greifswald, Rostock Germany; ^3^ Clinic for Psychosomatic and Psychotherapeutic Medicine, University Medicine Rostock, Rostock Germany; ^4^ Institut for Community Medicine, University Medicine, University of Greifswald, Greifswald Germany; ^5^ Deutsches Zentrum für Neurodegenerative Erkrankungen e. V. (DZNE), site Rostock / Greifswald, Greifswald, Mecklenburg Western Pomerania Germany; ^6^ Institute for Community Medicine / University of Greifswald, Greifswald Germany

## Abstract

**Background:**

The aim of the present study was twofold: First, to examine the validity of previously reported sociodemographic (age, sex) and clinical predictors (cognitive status, functional status, comorbidities) for the life expectancy in people with dementia in a community sample. Second, to investigate the role of social support beyond individual predictors.

**Method:**

The study utilizes data from 500 individuals living in the community who were diagnosed with dementia. These participants were monitored over a period of up to eight years. The research focused on assessing life expectancy in connection with established sociodemographic factors (such as age and sex), clinical predictors (including cognitive status, functional status, and comorbidities), and also considered the impact of social support. This was analyzed using Cox regression.

**Result:**

Advanced age increased the risk of mortality (hazard ratio [HR], 1.08; 95% CI, 1.05–1.11), while being female was associated with a lower mortality risk (HR, 0.65; 95% CI, 0.49–0.87). Higher cognitive (HR, 0.95; 95% CI, 0.93–0.98) and functional status at baseline (HR, 0.91; 95% CI, 0.86–0.97) were linked to increased life expectancy. Greater support from the social environment reduced the risk of mortality (HR, 0.79; 95% CI, 0.64–0.99), even after accounting for clinical variables.

**Conclusion:**

Previously reported sociodemographic and clinical predictors were confirmed within a community sample. Additionally, social support was found to predict life expectancy in individuals with dementia. Our findings highlight the crucial role of the social environment for the well‐being of individuals with dementia and emphasise the need for interventions that specifically address these factors.

